# Total Laparoscopic Excision of a Large Tailgut Cyst

**DOI:** 10.7759/cureus.71098

**Published:** 2024-10-08

**Authors:** Ajay Nimbalkar, Anand Zingade, Balaji Dhaigude

**Affiliations:** 1 General Surgery, Postgraduate Institute, Yashwantrao Chavan Memorial Hospital, Pimpri, Pune, IND

**Keywords:** congenital cyst, excision, laparoscopic, retrorectal cystic hamartomas, tailgut cysts

## Abstract

Tailgut cysts are rare congenital cysts that develop from the embryological remnants of the gut. They are usually found in the retrorectal space. In most of the cases, they are asymptomatic. The treatment of choice for tailgut cysts is complete surgical excision and both open and laparoscopic approaches have been described in the literature. The laparoscopic approach provides better visualization of pelvic structures and results in better post-operative outcomes when compared to the open approach. However, it can be challenging in cases with large tailgut cysts. We present a case of a female patient with a large tailgut cyst who underwent total laparoscopic excision.

## Introduction

Tailgut cysts, or retrorectal cystic hamartomas, are rare developmental lesions that occur congenitally. They are derived from incompletely regressed remnants of the embryonic postanal gut [1-2].

During the embryonic period, the embryo develops an appendage that is an extension of the embryonic hindgut, known as the tailgut. If the embryonic hindgut does not regress properly, it can result in the development of a tailgut cyst. However, in some cases, tailgut cysts are associated with the multiplication of meningothelial cells and the presence of benign thyroid tissue with oncocytic transition. As a result, there is disagreement regarding the aetiology of tailgut cysts [3].

Depending on the location of the tumor and the surgeon’'s preference, various approaches can be used to remove it, such as transperineal, sacrococcygeal, or abdominal [4].

Surgical resection is the preferred treatment method, with laparoscopic abdominal technique being recommended for surgeons proficient in pelvic laparoscopic surgery. This approach provides better visualization of pelvic viscera with lesser morbidity. However, if malignancy is suspected, the laparoscopic approach should not be performed due to the risk of rupture and seeding [5].

Large tailgut cysts can present a challenge during laparoscopic surgery. In this case, we present a female patient with a large tailgut cyst who underwent total laparoscopic excision.

## Case presentation

**Figure 1:**
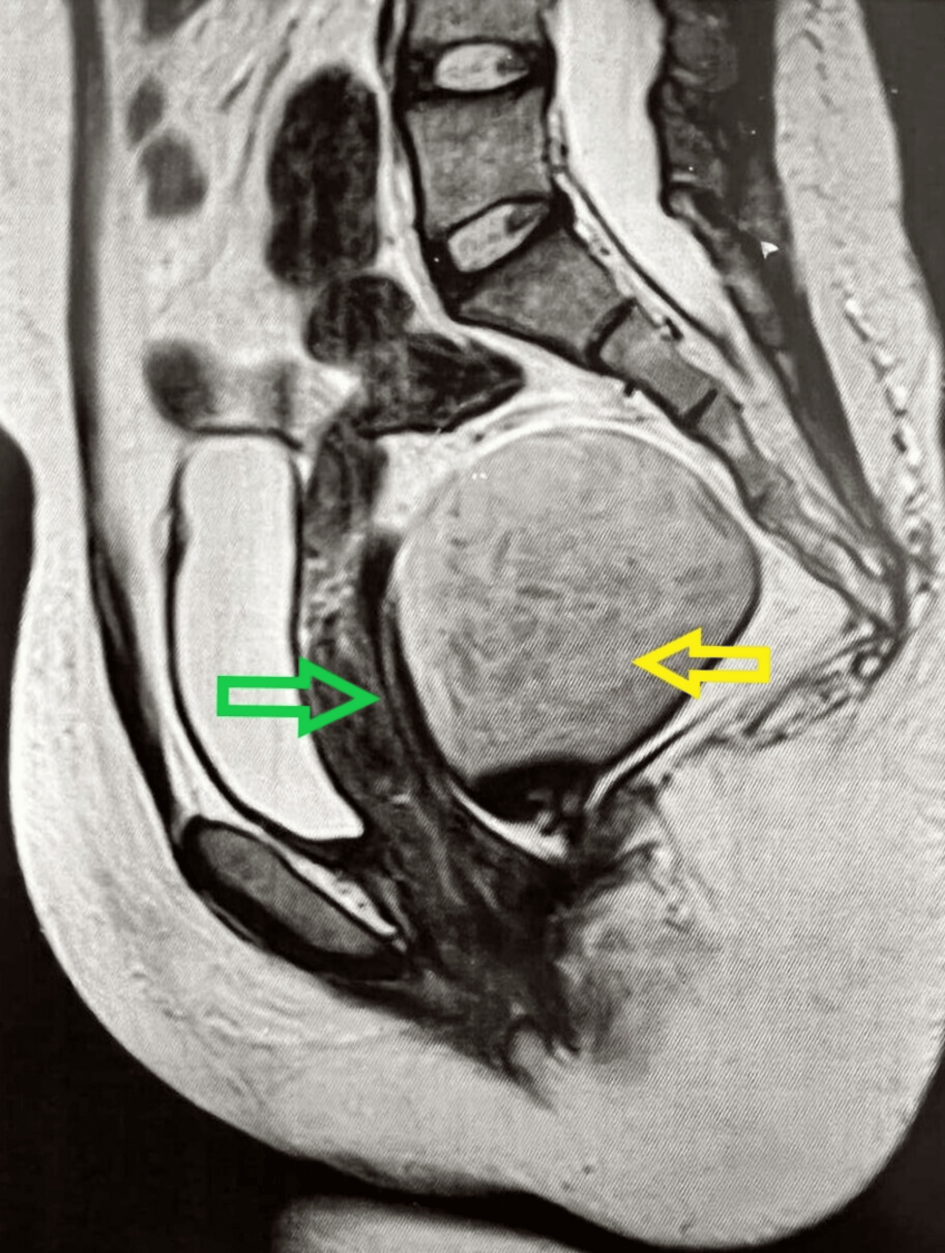
MRI of the pelvis showing tailgut cyst (yellow arrow) in the presacral space behind the rectum (green arrow)

A 27-year-old female presented with a history of a miscarriage and lower abdominal and perineal discomfort. Her general examination was normal and digital rectal examination revealed a lesion bulging from the posterior rectal wall. Her MRI (Magnetic Resonance Imaging) showed a well-defined cystic lesion of 10 × 8 cm × 8 cm in the retrorectal space. It displaced the rectum and uterus anteriorly. There was no evidence of any local invasion on MRI (Figure 1).

**Figure 2:**
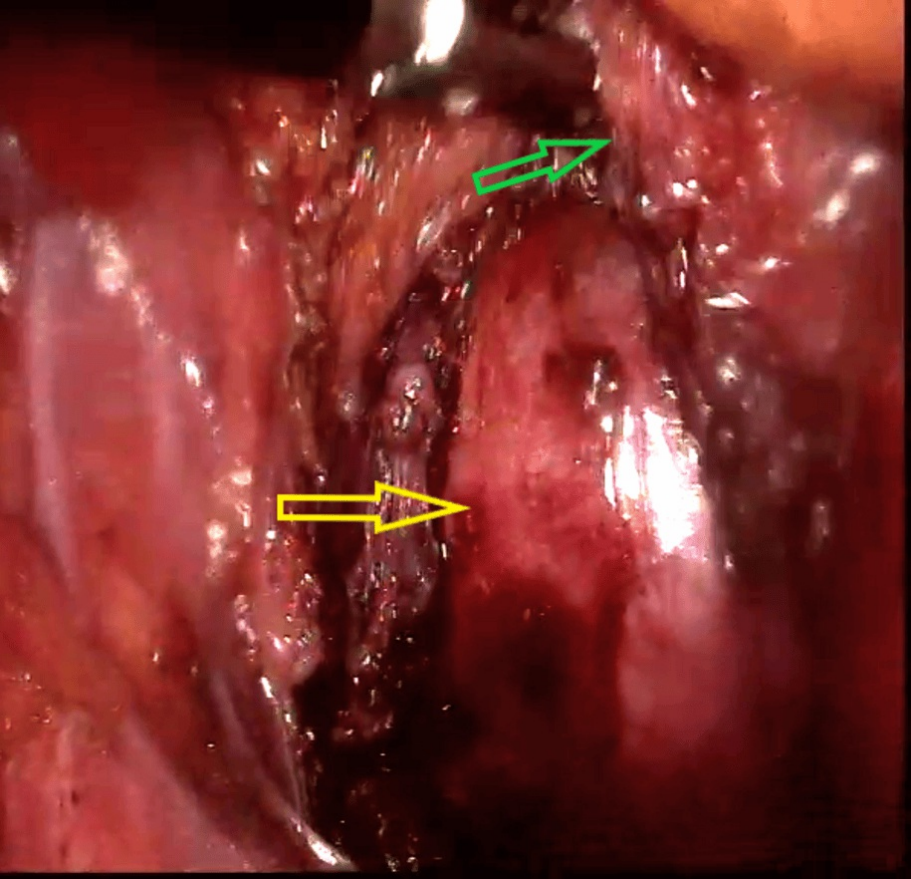
Laparoscopic dissection of the cyst wall (yellow arrow) from surrounding structures and rectum (green arrow)

**Figure 3:**
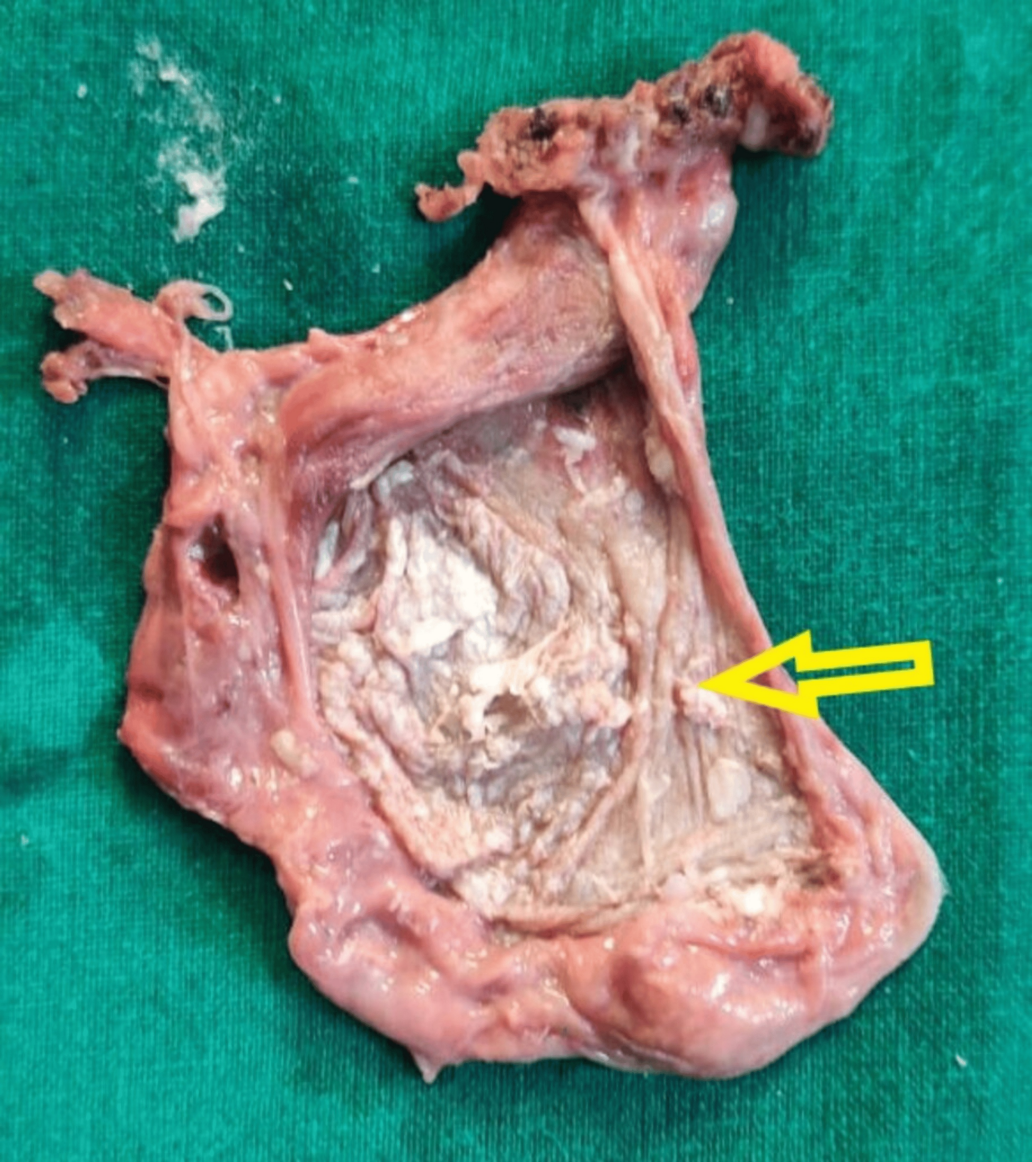
The cyst was cut open to reveal a thick cyst wall (yellow arrow) containing sebum-like material without any septations

Since the cyst appeared to be benign on the MRI, we opted for laparoscopic excision. The patient was positioned in lithotomy with a Trendelenburg position suitable for a digital rectal examination during the surgery. The procedure began with a 10 mm supraumbilical port insertion, followed by a 10 mm port in the right upper quadrant, a 5 mm port in the right lower quadrant, and a 5 mm port in the left upper and lower quadrant. The mesorectal dissection was performed from the sacral promontory up to the level of the puborectalis sling and the levator ani muscles, with careful dissection around the cyst wall (Figure 2). The tumor was resected completely and an intrabdominal drain was placed. Before removing the specimen, a leak test was performed to ensure the rectum was intact. The specimen was extracted in a specimen extraction bag. The cyst contained white, thick sebum-like material without any septations (Figure 3).

**Figure 4:**
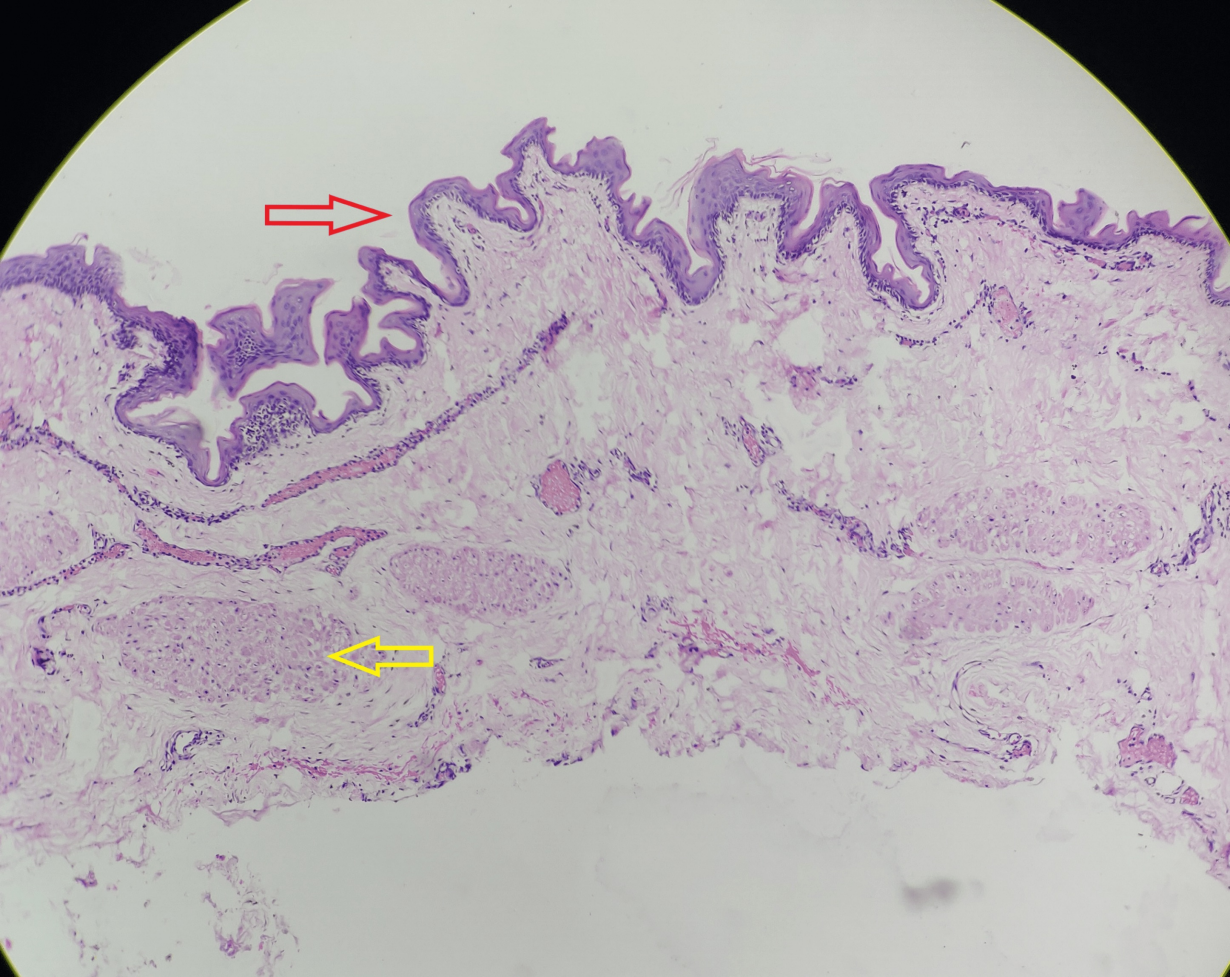
Histology on hematoxylin and eosin (H&E) stain and 10× magnification The histology shows the cyst wall with a keratinized stratified squamous epithelium (red arrow) and splaying of muscle fibers in the cyst wall (yellow arrow).

The patient was discharged on the second day after the surgery and was followed up for six months. The histopathology report confirmed the presence of a benign tailgut cyst. The cyst wall displayed splaying of muscle fibers, and the cyst contained a keratinized stratified squamous epithelium as observed in this slide (Figure 4).

## Discussion

These congenital cysts may present at any age, although they are most commonly diagnosed in individuals aged 30 to 60. Females are more prone to getting them than males, with a female-to-male ratio of 5:1. Most cysts do not cause any symptoms and are often missed during examination [6]. In 1984, Bale identified two cases of tailgut cysts out of 363 presacral masses, resulting in an incidence of 0.55% among sacrococcygeal lesions in children [7]. The estimated incidence of tailgut cysts is 1 in 40000 [8].

Tailgut cysts are usually discovered incidentally and the symptoms depend on their size and their relation to adjacent structures. Infection is the most common complication, occurring in 40-50% of cases. Although they are usually asymptomatic, patients with tailgut cysts may experience abdominal pain, rectal bleeding, local abscess formation, and rectal fullness or constipation [9].

Various techniques have been used to treat tailgut cysts. The utilization of a transabdominal approach ensures the comprehensive visualization of vital anatomical structures, thereby facilitating a more effective oncological resection in cases where malignancy is suspected. [10]. Recently, laparoscopic resection has been reported as a valid alternative to standard Kraske or other abdominal procedures. Potential benefits of the laparoscopic approach include fewer complications, faster recovery of bowel function, shorter hospital stays, less post-operative pain, reduced blood loss, and improved cosmetic outcomes [11]. However, it can be challenging in cases with large tailgut cysts.

Tailgut cysts have the potential to become malignant, such as adenocarcinoma, carcinoid, neuroendocrine carcinoma, or sarcoma. Hormones, particularly ghrelin and estrogen, are believed to have a significant influence on this transformation. The precise mechanism underlying this malignant transformation remains incompletely elucidated [3]. Therefore, complete surgical excision without spillage is necessary to prevent complications, such as infection, recurrence, and malignant transformation [9].

## Conclusions

In conclusion, the laparoscopic approach to tailgut cysts is feasible even for large-sized tailgut cysts. However, preoperative imaging is crucial to assess whether the cyst could be malignant or not, as there is a risk of spreading dysplastic cells if the cyst is accidentally opened. In such cases, it is recommended to consider an alternative approach based on the clinical findings and the surgeon's preference.
